# *Mycobacterium vaccae* immunization in rats ameliorates features of age-associated microglia activation in the amygdala and hippocampus

**DOI:** 10.1038/s41598-022-05275-y

**Published:** 2022-02-09

**Authors:** Kevin Sanchez, Jeffrey S. Darling, Reha Kakkar, Sienna L. Wu, Andrew Zentay, Christopher A. Lowry, Laura K. Fonken

**Affiliations:** 1 Division of Pharmacology and Toxicology, College of Pharmacy, The University of Texas at Austin, 107 W Dean Keeton St 3.510C, Austin, TX 78712 USA; 2 Department of Integrative Physiology, University of Colorado Boulder, Boulder, CO 80309 USA; 3 Center for Neuroscience, University of Colorado Boulder, Boulder, CO 80309 USA

**Keywords:** Microglia, Neuroimmunology

## Abstract

Aging and reduced exposure to environmental microbes can both potentiate neuroinflammatory responses. Prior studies indicate that immunization with the immunoregulatory and anti-inflammatory bacterium, *Mycobacterium vaccae* (*M. vaccae*), in aged rats limits neuroimmune activation and cognitive impairments. However, the mechanisms by which *M. vaccae* immunization ameliorates age-associated neuroinflammatory “priming” and whether microglia are a primary target remain unclear. Here, we investigated whether *M. vaccae* immunization protects against microglia morphological changes in response to aging. Adult (3 mos) and aged (24 mos) Fisher 344 × Brown Norway rats were immunized with either *M. vaccae* or vehicle once every week for 3 weeks. Aging led to elevated Iba1 immunoreactivity, microglial density, and deramification of microglia processes in the hippocampus and amygdala but not other brain regions. Additionally, aged rats exhibited larger microglial somas in the dorsal hippocampus, suggestive of a more activated phenotype. Notably, *M. vaccae* treatment ameliorated indicators of microglia activation in both the amygdala and hippocampus. While changes in morphology appeared to be region-specific, gene markers indicative of microglia activation were upregulated by age and lowered in response to *M. vaccae* in all brain regions evaluated. Taken together, these data suggest that peripheral immunization with *M. vaccae* quells markers of age-associated microglia activation.

## Introduction

The proportion of aged individuals across the world is rising rapidly; the United Nations projects the number of people over the age of 65 to double by 2050 to over two billion individuals^1^. The lengthening of the human lifespan is associated with a rise in the burden of age-associated neurological disorders. Indeed, the aging process is characterized by a progressive shift from a homeostatic balance of inflammatory markers towards a “primed” or sensitized state^2^. This increased neuroinflammatory priming makes the aged brain further susceptible to the disruptive effects of intrinsic and extrinsic factors like disease, infection, and stress^3–6^, thereby elevating the risk of affective disorders, cognitive impairments, and neurodegenerative diseases in the aged population^7, 8^.

In addition to aging, chronic inflammatory conditions are increasing. Elevated chronic low-grade inflammation among modern urban societies may be caused by decreased microbial exposures^9^—this is the foundation for the “Old Friends” hypothesis^9–13^. Throughout evolution, the mammalian immune system developed tolerance to commensal environmental microbes. One such example is *Mycobacterium vaccae* (*M. vaccae*), a saprophytic bacterium found in soil, water, and mud that our ancestors frequently encountered^14^. Reintroduction of these microbes in an excessively “clean” environment can suppress immune sensitization and reduce the risk for inflammatory diseases (reviewed by Lowry et al.^15^). *M. vaccae* has immunoregulatory properties, such as enhancing the induction of regulatory T cells and stimulating their production of anti-inflammatory cytokines, including interleukin (IL)-10 and transforming growth factor β^16, 17^. Peripheral immunization with *M. vaccae* also promotes an anti-inflammatory milieu in the central nervous system (CNS)^18–20^.

Elevated neuroinflammatory priming, as is observed due to aging, is mediated in part by microglia, the primary immunocompetent cell in the CNS. Microglia are dynamic cells that take on an array of phenotypes based on signals from their surrounding microenvironment. Under quiescent conditions, microglia communicate constantly with neurons via signaling dyads that maintain them in a surveillant state (e.g., CX3CL1:CX3CR1, CD200:CD200R)^21^. Morphologically, these microglia exhibit small, round somas with highly ramified processes that facilitate their communication with other CNS cell types^22^. When microglia detect adverse signals or molecules, their morphology can drastically change—microglial processes retract and thicken while their somas swell and become irregular^23, 24^. This morphology is associated with chemotaxis, secretion of pro-inflammatory cytokines such as IL-1β and tumor necrosis factor α, and phagocytosis.

Here, we investigate whether aging-related shifts in microglial morphology are ameliorated by immunization with anti-inflammatory *M. vaccae*. Morphological features of microglia evaluated in the amygdala, hippocampus, hypothalamus, and prefrontal cortex of adult (3 mos) and aged (24 mos) male Fisher 344 × Brown Norway rats. Our results demonstrate that aging leads to differential changes in microglia morphology and reactivity across brain regions, with the hippocampus being the most sensitive. Moreover, microglia in the amygdala and hippocampus appear most responsive to the anti-inflammatory effects of *M. vaccae* immunization, protecting against some age-associated microglia morphological changes.

## Results

### Aging modulates Iba1 immunoreactivity and density in a region-specific manner

**Figure 1:**
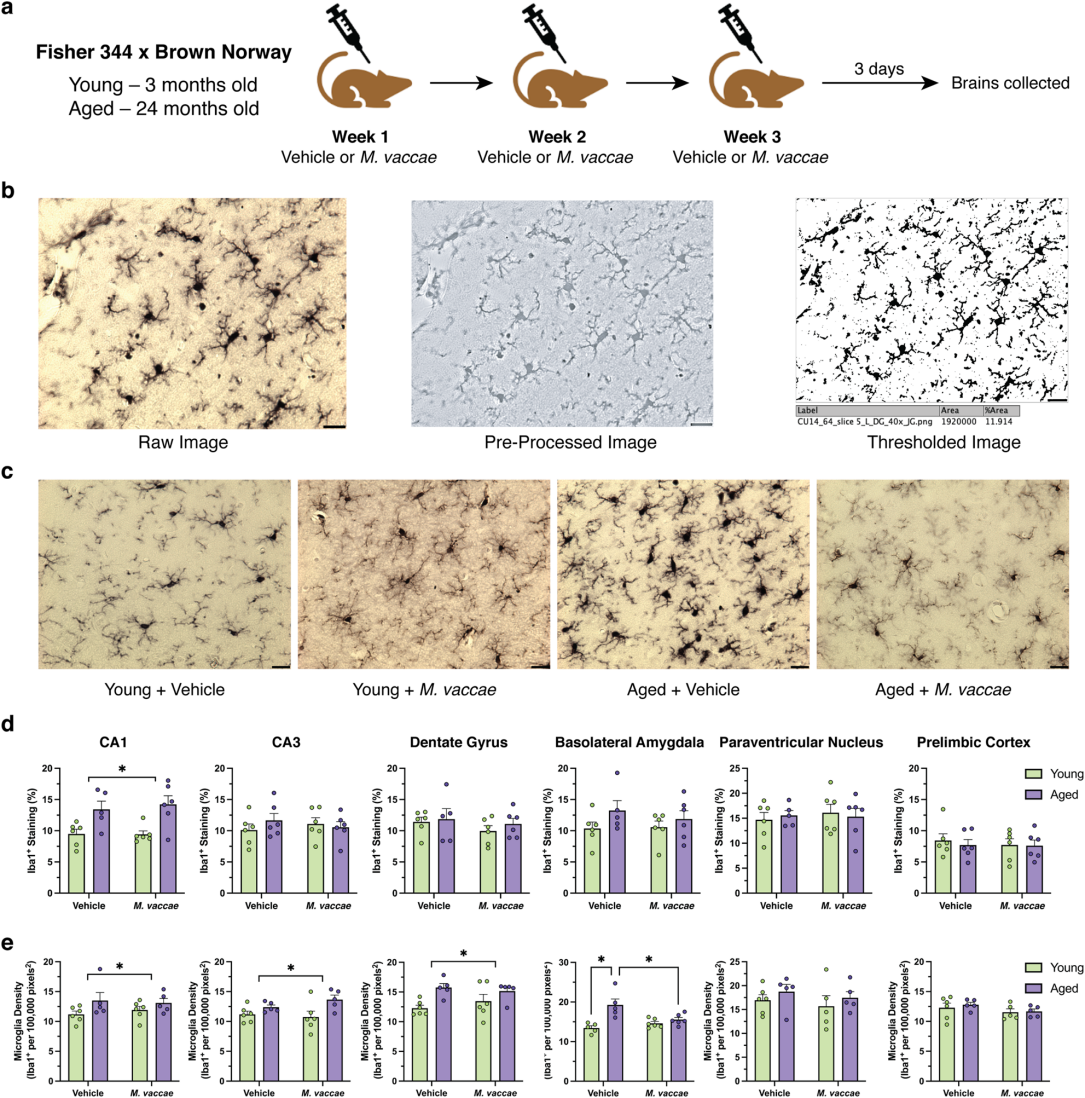
Microglial area fraction and density were elevated in the hippocampus and basolateral amygdala with aging, but only ameliorated by *M. vaccae* immunization in the amygdala. (**a**) Experimental timeline illustrating the timing of the *M. vaccae* immunizations. A dose of 100 μg of *M. vaccae* was administered subcutaneously once a week for three weeks. Three days after the final injection, brains were collected and perfused for subsequent microglia morphological analyses. (**b**) Methodology for performing area fraction analysis. Raw images from Iba1-stained slides were first pre-processed using a macro on FIJI to remove background noise and enhance clarity of microglia. This image was then thresholded and area fraction analysis performed. (**c**) Representative images from the CA3 subfield for each experimental group indicate differences in soma size and branching due to aging and *M. vaccae* immunization. (**d**,**e**) Area fraction and microglia density results for the basolateral amygdala, CA1, CA3, dentate gyrus, paraventricular nucleus, and prelimbic cortex. Iba1^+^ staining was the percent area containing the diaminobenzidine reaction product, and microglia density reflects normalized counts of the cell bodies in that brain region. The data are graphed as mean ± SEM; *n* = 5–6 rats/group; scale bar = 20 μm; *p < 0.05 (Tukey’s post hoc test) or *p* < 0.05, main effect of age.

Aging is associated with a shift towards an increase in microglia priming^25^. Here, we examine whether *M. vaccae* immunization ameliorated features indicative of microglia priming/activation, including Iba1 immunoreactivity and microglia density, in the aged rat brain. Three days following the final *M. vaccae* immunization, brains were collected, sliced, and stained with diaminobenzidine (DAB) against Iba1 to visualize microglia (Fig. 1a). Area fraction analysis was performed to assess whether aging or *M. vaccae* immunization led to an overall change in density of Iba1 immunoreactivity^26^. Iba1 immunoreactivity is often used as a first pass to assess gross changes in microglia morphology; however, it does not detect subtle changes. Images were first pre-processed to remove background noise before being thresholded for calculating the area fraction (Fig. 1b). Representative images from the CA3 hippocampal subfield (Fig. 1c) and all other brain regions (Supplementary Fig. S1) suggest differences in microglia soma size and branching complexity across experimental groups. Aging, but not *M. vaccae*, altered Iba1 immunoreactivity in the CA1 subfield, such that Iba1 occupied a larger percentage of the image (main effect age, *F*_(1,19)_ = 17.57, *p* < 0.05; Fig. 1d). No age- or *M. vaccae*-associated differences in thresholding of Iba1 immunoreactivity were detected in other brain regions, including the CA3, DG, BLA, PrL, and PVN (*p* > 0.05; Fig. 1d).

Interestingly, although changes in immunoreactivity were only detected in the CA1, there were age-associated changes in microglia density (i.e., number of microglia per area) in several brain regions. Aged rats had elevated density of microglia in the BLA (interaction effect, *F*_(1,18)_ = 8.833, *p* < 0.05; post hoc young vehicle vs. aged vehicle, *p* < 0.05; Fig. 1e), CA1 (main effect age, *F*_(1,18)_ = 4.463, *p* < 0.05), CA3 (main effect age, *F*_(1,18)_ = 8.053, *p* < 0.05), and DG (main effect age, *F*_(1,18)_ = 9.834, *p* < 0.05). The age-associated increase in microglia density was ameliorated by *M. vaccae* in the BLA (interaction effect, *F*_(1,18)_ = 8.833, *p* < 0.05; post hoc aged vehicle vs. aged *M. vaccae*, *p* < 0.05) but not other brain regions (*p* > 0.05; Fig. 1e). Importantly, neither area fraction nor microglia density analyses capture changes in microglia morphology and localization of these alterations (e.g., soma, branches), which may underlie why changes in density and immunoreactivity occurred in distinct brain regions. Thus, we next performed microglia morphological analyses for soma characteristics and branching complexity.

### Microglial somas in the hippocampus appear more vulnerable to the effects of aging and *M. vaccae* immunization

**Figure 2:**
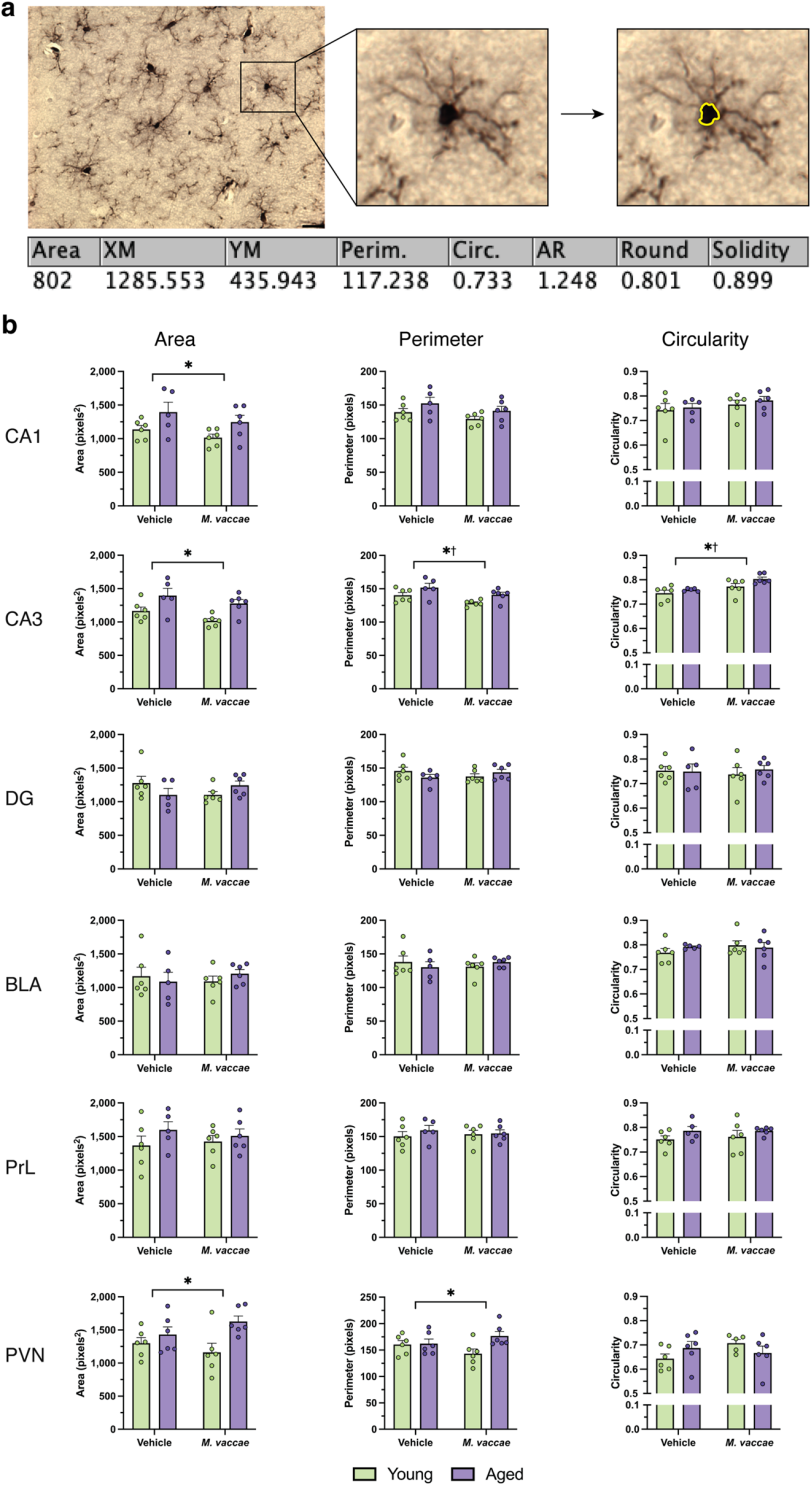
Microglia somas from the CA3 hippocampal subfield are responsive to the effects of both aging and *M. vaccae* immunization. (**a**) Methodology for assessing microglia soma morphology. Somas were traced using a graphic design tablet and analyzed using FIJI to automatically generate its area, perimeter, and circularity. (**b**) Values for the area, perimeter, and circularity of the microglial soma in the basolateral amygdala, CA1, CA3, dentate gyrus, paraventricular nucleus, and prelimbic cortex. The area was calculated in pixels squared based on the traced soma, and the perimeter was the number of pixels outlining that same soma. Circularity was determined to be $\frac{4\pi A}{{P}^{2}}$, where *A* is area and *P* is perimeter. A value of 1.0 represents a perfect circle, whereas an increasingly elongated shape has a circularity approaching 0.0. The data are graphed as mean ± SEM; *n* = 5–6 rats/group (12 microglia/rat); scale bar = 20 μm; *p < 0.05, main effect of age; ^†^*p* < 0.05, main effect of *M. vaccae*.

In response to inflammatory challenge, microglial somas increase in area and decrease in circularity^23^; however, it remains unclear the effect that aging has on the morphology of the microglial soma in F344 x BN rats. To assess this, microglial somas were analyzed for area, perimeter, and circularity (Fig. 2a). No differences in soma area, perimeter, or circularity were apparent in the BLA, DG, or PrL (*p* > 0.05; Fig. 2b). In contrast, several morphological changes in microglia somas were detected in the PVN and CA subfields of the dorsal hippocampus. Microglia in the PVN of aged rats had a larger area (main effect age, *F*_(1,20)_ = 7.683, *p* < 0.05) and perimeter (main effect age, *F*_(1,20)_ = 4.411, *p* < 0.05) than young rats. In the CA1 hippocampal subfield, there was an age-related elevation in CA1 microglia soma area (main effect age, *F*_(1,19)_ = 7.258, *p* < 0.05). Moreover, in the CA3, age and *M. vaccae* immunization altered microglial soma characteristics. Microglia in the CA3 of the aged rat brain had a greater area (main effect age, *F*_(1,19)_ = 13.35, *p* < 0.05), perimeter (main effect age, *F*_(1,19)_ = 8.443, *p* < 0.05), and circularity (main effect age, *F*_(1,19)_ = 4.641, *p* < 0.05). Immunization with *M. vaccae* decreased CA3 microglia perimeter (main effect *M. vaccae*, *F*_(1,19)_ = 7.548, *p* < 0.05) and further increased circularity (main effect *M. vaccae*, *F*_(1,19)_ = 11.57, *p* < 0.05; Fig. 2b) in both young and aged rats. This indicates that *M. vaccae* immunization may promote morphological changes consistent with a less activated microglia phenotype in the CA3 hippocampal subfield.

### *M. vaccae* immunization has differential age-dependent effects on microglial branching in the amygdala and hippocampus

**Figure 3:**
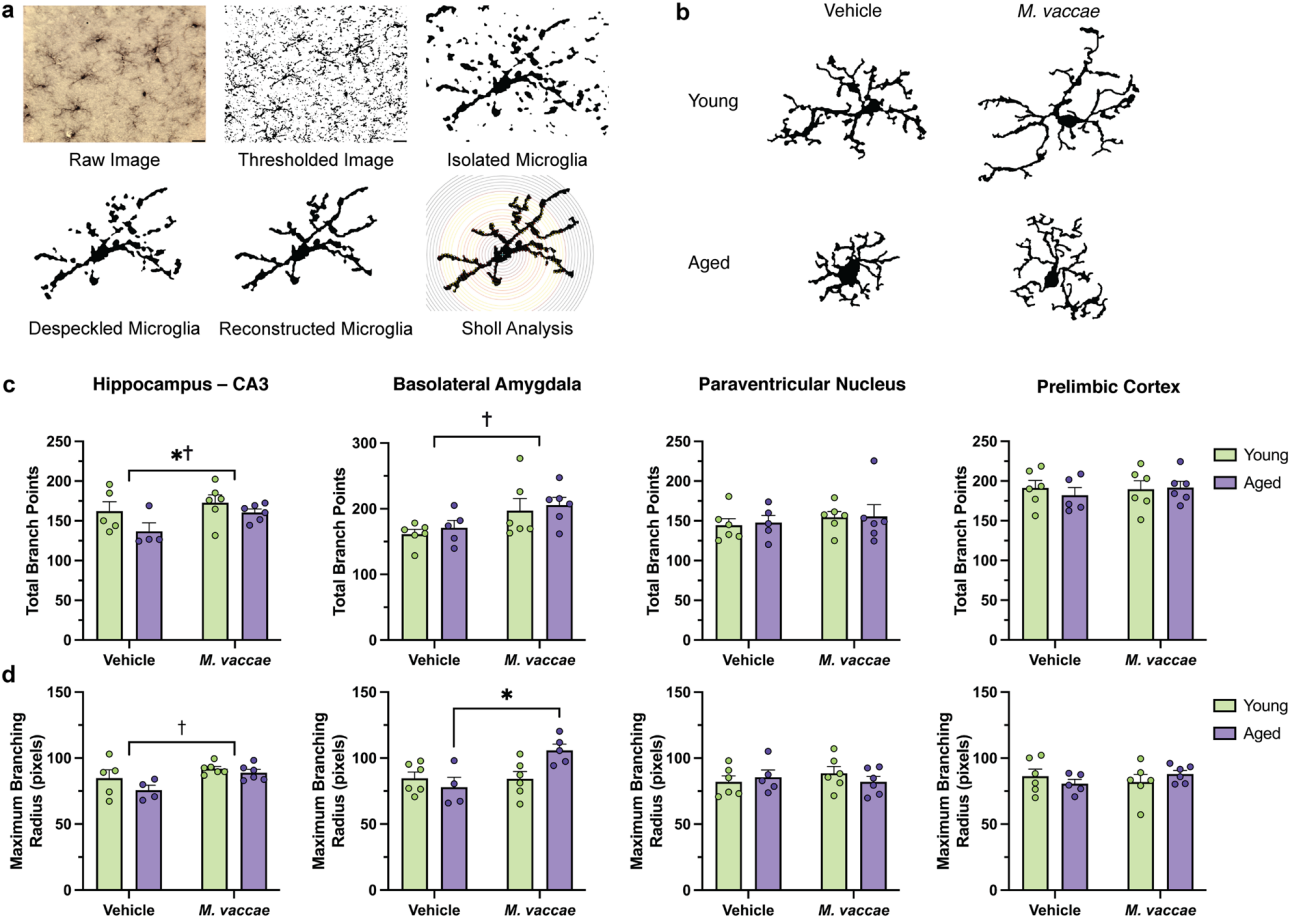
*M. vaccae* immunization induces microglial branching in the basolateral amygdala and CA3 hippocampal subfield of aged rats. (**a**) Methodology for performing a Sholl analysis. Microglia were thresholded, despeckled to remove background noise, and manually reconstructed while referring to the original image for accuracy. The Sholl analysis is then performed with concentric circles appearing at a starting radius of five pixels from the soma, and the step size was set at seven pixels. (**b**) Representative images of the Sholl analysis (to scale) demonstrating differences in microglial complexity across each group. (**c**) Total branch points were determined as the sum of all intersections between microglial processes and concentric circles. (**d**) Maximum branching radius was the distance where the highest count of intersections occurred. This reflects sites with the greatest branch density. The data are graphed as mean ± SEM; *n* = 4–6 rats/group (3–5 microglia/rat); scale bar = 20 μm; *p < 0.05 (Tukey’s post hoc test) or *p* < 0.05, main effect of age; ^†^*p* < 0.05, main effect of *M. vaccae*.

We next investigated the effects of aging and *M. vaccae* immunization on microglial branching and complexity in the BLA, CA3, PVN, and PrL. The CA3 was selected as a representative region of the hippocampus over the CA1 and DG since *M. vaccae* immunization led to the most robust change in the CA3 (Fig. 3a). The total number of intersections between the concentric circles emanating from the microglia soma and its processes (i.e., total branch points) were decreased in the CA3 with age (main effect age, *F*_(1,17)_ = 5.406, *p* < 0.05; Fig. 3c). This decrease was rescued by *M. vaccae* immunization in the CA3 (main effect *M. vaccae*, *F*_(1,17)_ = 6.461, *p* < 0.05; Fig. 3b,c). Furthermore, *M. vaccae* immunization also increased branching in the BLA, although there was no baseline effect of age (main effect *M. vaccae*, *F*_(1,19)_ = 7.307, *p* < 0.05; Fig. 3c). Additionally, the radius where maximum branching occurred was greater in the CA3 after *M. vaccae* (main effect *M. vaccae*, *F*_(1,17)_ = 7.201, *p* < 0.05; Fig. 3d), such that processes were located more distal to the soma, suggestive of less activated and more surveillant microglia. The maximum branching radius was also elevated by *M. vaccae* immunization in the BLA, specifically in aged rats (interaction effect, *F*_(1,17)_ = 6.485, *p* < 0.05; post hoc aged vehicle vs. aged *M. vaccae*, *p* < 0.05; Fig. 3d).

**Figure 4:**
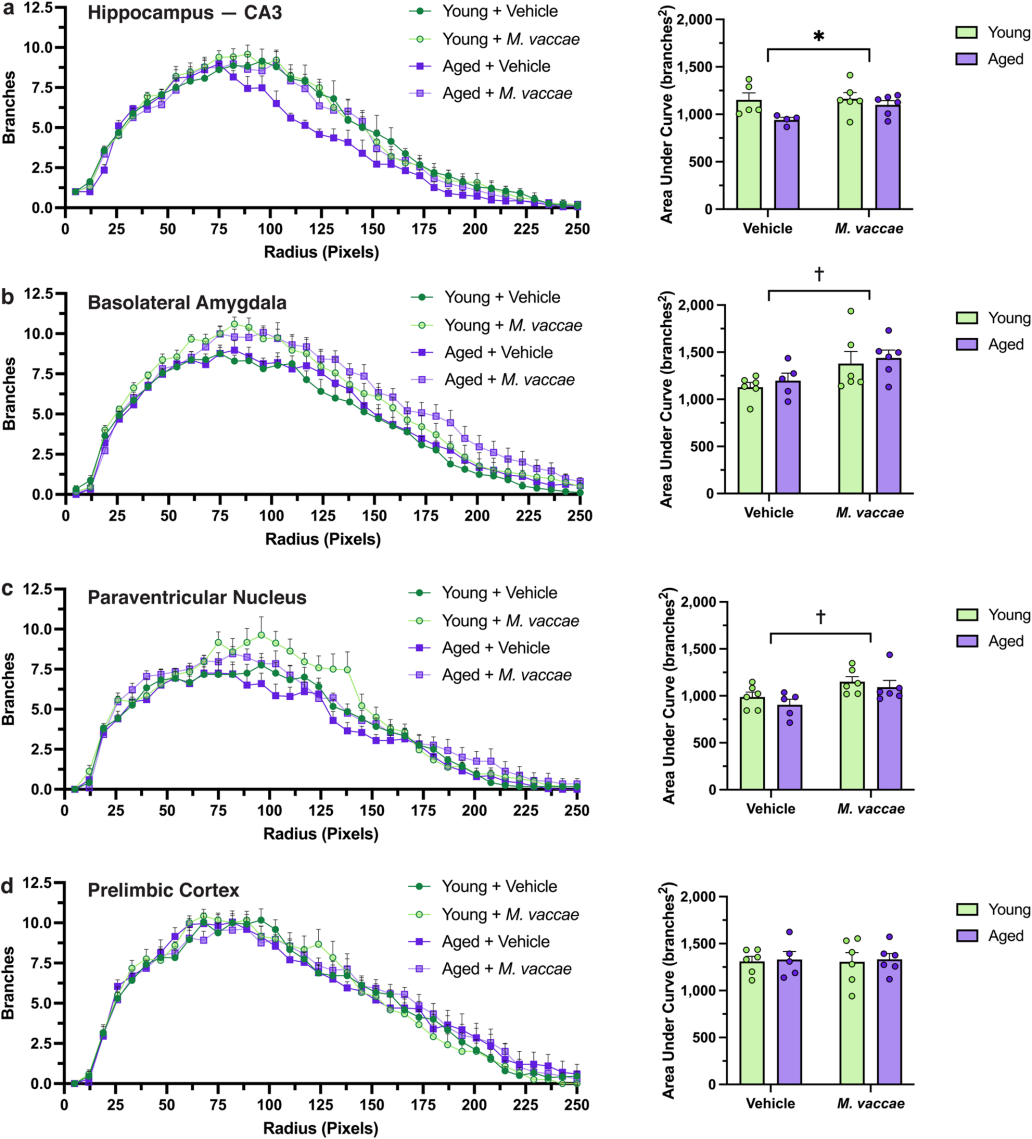
*M. vaccae* immunization leads to elevated overall branching independent of age in the basolateral amygdala and paraventricular nucleus. (**a**–**d**) Plotting branches vs. radius from soma reveals differential changes in microglial branching due to age and *M. vaccae* immunization. Global differences in microglial complexity were assessed with an area under the curve analysis. The data are graphed as mean ± SEM; *n* = 4–6 rats/group (3–5 microglia/rat); *p < 0.05, main effect of age; ^†^*p* < 0.05, main effect of *M. vaccae*.

We subsequently analyzed microglial branching across all distances from the soma using an area under curve analysis. In the CA3 subfield, aged rats administered vehicle had reduced branching compared to their younger counterparts (main effect age, *F*_(1,17)_ = 5.373, *p* < 0.05; Fig. 4a). Interestingly, *M. vaccae* immunization promoted branching in both the BLA (main effect *M. vaccae*, *F*_(1,19)_ = 40.8, *p* < 0.05; Fig. 4b) and PVN (main effect *M. vaccae*, *F*_(1,19)_ = 8.627, *p* < 0.05; Fig. 4c). No difference in overall branching was noted in the PrL due to age or *M. vaccae* immunization (*p* > 0.05; Fig. 4d). Taken together, our results from the Sholl analysis suggest that the BLA and CA3 may be the most responsive to *M. vaccae* immunization. However, *M. vaccae* may also be effective in other brain regions, such as the PVN, as evidenced by an overall increase in branching.

### Skeletonized microglia have increased branching in the amygdala and hippocampus following *M. vaccae* immunization

**Figure 5:**
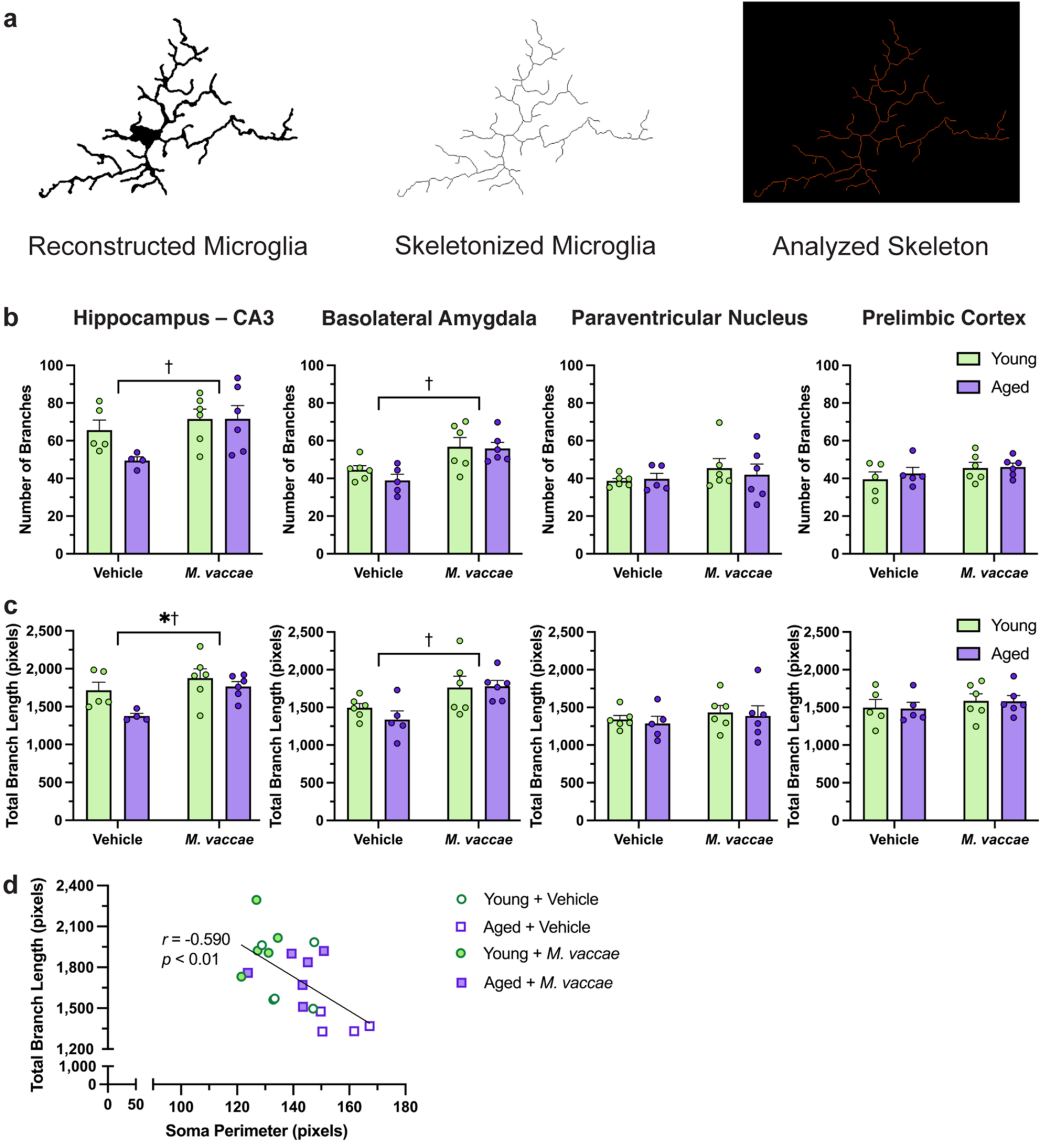
Skeletonization analysis suggest that microglia from the basolateral amygdala and hippocampus are responsive to *M. vaccae* immunization. (**a**) Methodology for performing the skeletonization analysis. Reconstructed microglia from the Sholl analysis were skeletonized and processed using a plug-in on FIJI. (**b**) The number of branches were calculated for each skeleton. A branch is considered a continuous segment that connects endpoints, endpoints and junctions, or junctions and junctions. (**c**) The total branch length was computed for each skeleton. This is the sum of each individual pixel in a skeletonized image. (**d**) Linear regression analysis between total branch length and soma perimeter from the hippocampus reveals a negative correlation. The data are graphed as mean ± SEM; *n* = 4–6 rats/group; *p < 0.05, main effect of age; †*p* < 0.05, main effect of *M. vaccae*.

After the Sholl analysis, we skeletonized reconstructed microglia by using the Skeletonize3D plug-in on FIJI (Fig. 5a). This was followed by the Analyze Skeleton 2D/3D plug-in to obtain data on the number of branches and total branch length. *M. vaccae* immunization led to a greater number of branches in both the BLA (main effect *M. vaccae*, *F*_(1,19)_ = 16.28, *p* < 0.05; Fig. 5b) and CA3 subfield (main effect *M. vaccae*, *F*_(1,17)_ = 5.855, *p* < 0.05), paralleling the results from the Sholl analysis. Rats immunized with *M. vaccae* also had increases in total branch length in the BLA (main effect *M. vaccae*, *F*_(1,19)_ = 11.09, *p* < 0.05; Fig. 5c) and CA3 (main effect *M. vaccae*, *F*_(1,17)_ = 7.935, *p* < 0.05). Moreover, microglia in the aged rat CA3 also had decreased total branch length compared to young rats (main effect age, *F*_(1,17)_ = 5.257, *p* < 0.05; Fig. 5c).

Total microglial branch length from the CA3 hippocampal subfield was then plotted against the soma perimeter to establish whether these morphological indicators of microglial reactivity were co-occurring. We expected that rats that demonstrate greater microglial branching would also have a smaller soma perimeter since these features are indicative of a quiescent phenotype. A negative correlation was observed between these two variables (*r* = − 0.590, *F*_(1,18)_ = 9.619, *p* < 0.05; Fig. 5d). Among rats that received vehicle injections, young rats tended to cluster differently on the XY plane than aged rats (*χ*^2^ = 2.776, *p* = 0.09); specifically, aged rats tended to have less branching and larger somas compared to young rats. Furthermore, aged rats immunized with *M. vaccae* trended towards having greater branching and smaller somas compared to aged rats that received vehicle (*χ*^2^ = 3.546, *p* = 0.06; Fig. 5d). Thus, microglial branch length negatively correlated with soma size, suggesting that these microglial reactivity indicators occur concomitantly.

### Aged rats have elevated expression of microglia reactivity markers in the hippocampus, hypothalamus, and prefrontal cortex

**Figure 6:**
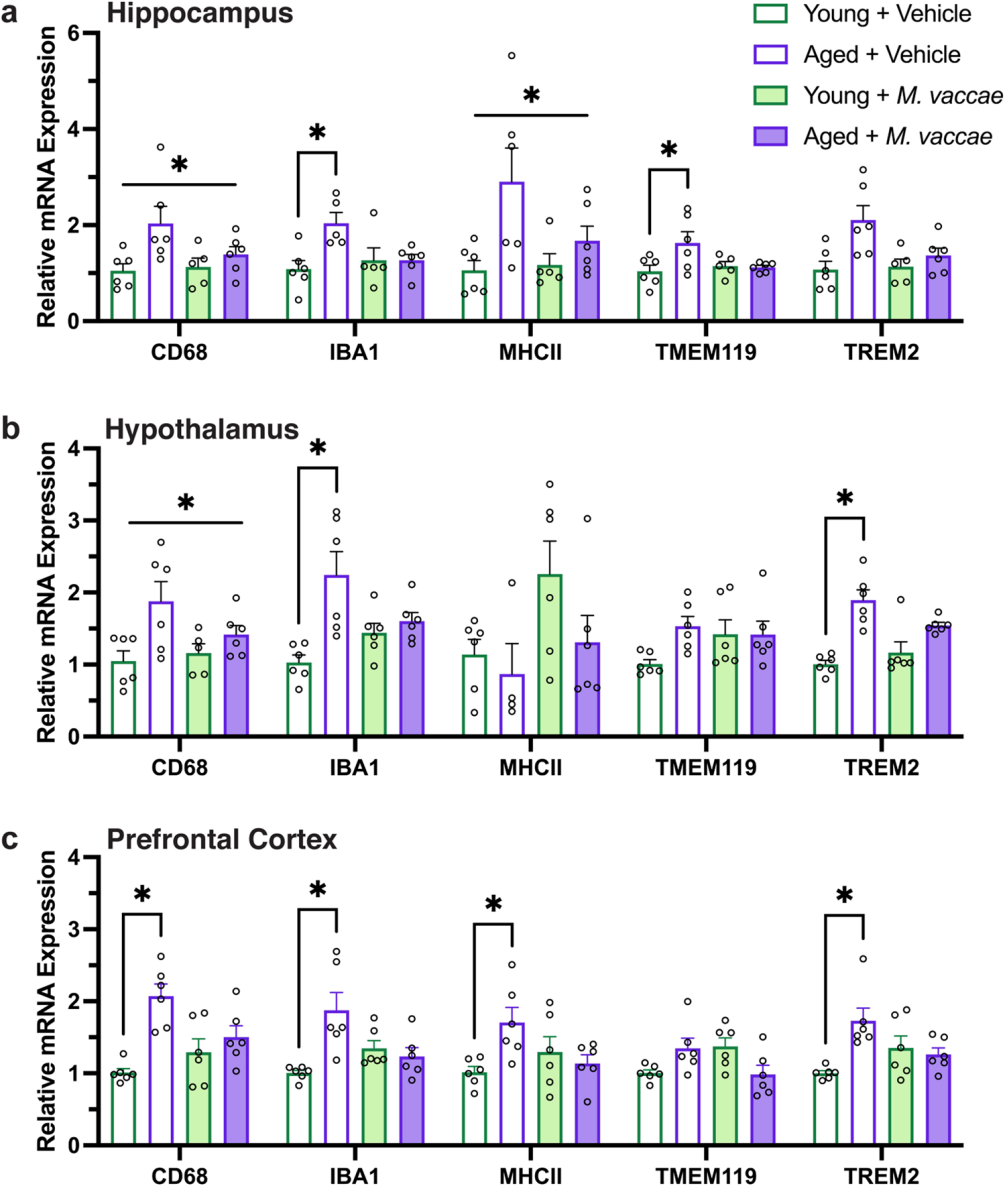
Aging results in elevated expression of microglia reactivity markers that are partly lowered by *M. vaccae* immunization. (**a**–**c**) Expression of microglia markers CD68, IBA1, MHCII, TMEM119, and TREM2 from the hippocampus, hypothalamus, and prefrontal cortex are generally elevated with age and partly reduced by *M. vaccae* immunization. The data are graphed as mean ± SEM; *n* = 4–6 rats/group; *p < 0.05 (Tukey’s post hoc test) or *p* < 0.05, main effect of age.

Given our microglia morphological results, we next sought to determine whether the expression of microglia markers was altered in response to aging and *M. vaccae* immunization. We investigated five microglia markers: CD68, IBA1, MHCII, TMEM119, and TREM2. CD68 is a microglia marker that is upregulated in primed microglia and during an inflammatory response to promote phagocytosis^27, 28^. Aged rats had greater expression of CD68 in the hippocampus (main effect age, *F*_(1,19)_ = 7.183, *p* < 0.05; Fig. 6a) and hypothalamus (main effect age, *F*_(1,19)_ = 8.609, *p* < 0.05; Fig. 6b) regardless of *M. vaccae* immunization. Aged rats also exhibited elevated prefrontal cortex expression of CD68 (interaction effect, *F*_(1,20)_ = 8.007, *p* < 0.05; post hoc young vehicle vs. aged vehicle, *p* < 0.05; Fig. 6c) that was ameliorated by *M. vaccae* immunization.

IBA1 is a calcium binding protein that is constitutively expressed in the brain by microglia^29^. IBA1 was ubiquitously elevated in aged rats that received vehicle injections in the hippocampus (interaction effect, *F*_(1,18)_ = 6.055, *p* < 0.05; post hoc young vehicle vs. aged vehicle, *p* < 0.05), hypothalamus (interaction effect, *F*_(1,20)_ = 7.435, *p* < 0.05; post hoc young vehicle vs. aged vehicle, *p* < 0.05), and prefrontal cortex (interaction effect, *F*_(1,20)_ = 10.90, *p* < 0.05; post hoc young vehicle vs. aged vehicle, *p* < 0.05). Immunization with *M. vaccae* in aged rats led to reductions in IBA1 expression to levels comparable to young rats.

MHCII is involved in antigen presentation is upregulated with microglia activation; surveillant microglia express lower MHCII expression compared to primed or activated microglia^28^. Aged rats had greater expression of MHCII (main effect age, *F*_(1,19)_ = 7.476, *p* < 0.05) in the hippocampus regardless of *M. vaccae* immunization. However, in the prefrontal cortex, there was elevated MHCII in aged rats receiving vehicle treatment (interaction effect, *F*_(1,20)_ = 6.622, *p* < 0.05; post hoc young vehicle vs. aged vehicle, *p* < 0.05) that was reduced by *M. vaccae*.

TMEM119 is a microglia-specific transmembrane marker that is commonly used to discriminate between microglia and blood-derived macrophages in the brain^30, 31^. Our results indicate that TMEM119 was upregulated only in the hippocampus of aged rats (interaction effect, *F*_(1,19)_ = 4.507, *p* < 0.05; post hoc young vehicle vs. aged vehicle, *p* < 0.05; Fig. 6a). *M. vaccae* immunization in aged rats lowered TMEM119 expression to levels similar to young rats.

Lastly, TREM2 is a microglial marker that has important roles in microglia phagocytosis of apoptotic neurons, damaged myelin, and amyloid plaques^32^. Consistent with prior work^33, 34^, aged rats that received vehicle injections had increased mRNA expression of TREM2 relative to their younger counterparts in the hypothalamus (interaction effect, *F*_(1,20)_ = 5.528, *p* < 0.05; post hoc young vehicle vs. aged vehicle, *p* < 0.05; Fig. 6b) and prefrontal cortex (interaction effect, *F*_(1,20)_ = 9.739, *p* < 0.05; post hoc young vehicle vs. aged vehicle, *p* < 0.05; Fig. 6c) that was reduced in response to *M. vaccae*. Collectively, these data indicate that aging is associated with upregulation of markers reflective of microglial reactivity across the hippocampus, hypothalamus, and prefrontal cortex. Moreover, *M. vaccae* immunization partially lowered the expression of these microglial markers across these three brain regions in aged rats.

## Discussion

Aging is associated with a rise in chronic low-grade neuroinflammation or “inflammaging” that increases susceptibility to cognitive impairments and neurodegenerative diseases^7, 8^. This increased neuroinflammation is primarily mediated by microglia, and their morphology is one indicator of their activation state^35^. Here, we investigated whether subcutaneous *M. vaccae* immunization mitigated age-associated changes in microglia morphology and gene expression markers. Our results suggest that aging alters microglia morphology in a region-specific manner, with the hippocampus being most susceptible to these changes. Moreover, microglia in the amygdala and hippocampus were most responsive to the anti-inflammatory effects conferred by *M. vaccae* immunization. Although morphological changes were region specific, microglia associated gene changes occurred across brain regions with aging and *M. vaccae* immunization. Aged rats had elevated mRNA expression of microglia-specific markers in the hippocampus, hypothalamus, and prefrontal cortex that were partly reduced by *M. vaccae*. Altogether, these data indicate that whereas microglia from the hippocampus were most sensitive to the effects of aging, *M. vaccae* specifically targeted microglia morphology promoting a less activated phenotype only in the amygdala and hippocampus.

With aging, a subset of microglia acquires a dystrophic or senescent phenotype that impairs their ability to appropriately respond to stimuli^36, 37^. Dystrophic microglia in the aged brain have numerous morphological aberrations including soma hypertrophy accompanied by deramified, beaded, and discontinuous processes^38^. Our findings further support microglia morphological changes with aging. For example, in the CA3 hippocampal subfield, there were age-associated increases in microglia density and soma size that were accompanied with decreased branching. These morphological features from our study correlate with reactive microglia that have elevated uptake of immunomodulatory proteins, secretion of cytotoxic factors, and release of inflammatory molecules that stimulate pro-inflammatory signaling cascades^39^.

The hippocampus was the most sensitive to age-associated changes in microglia morphology across all studied regions. Microglia from the hippocampus of aged rats had elevated Iba1 immunoreactivity and microglia density accompanied by larger somas and deramified processes (i.e., reduced branching). This supports observations that suggest regional vulnerability in response to aging. For example, microglia from the hippocampus of aged mice survey their surrounding microenvironment less so than microglia from other forebrain regions like the cortex^40^. Hippocampal microglia also have potentiated expression of pro-inflammatory cytokines (e.g., *Il1b*) after an immune stimulus in comparison to other brain regions like the hypothalamus and cerebellum^41^. Indeed, our results indicate that aged rats have increased expression of hippocampal CD68 and MHCII, consistent with a more “primed” immune environment in the aged brain^42^. We also noted upregulation of IBA1 in aged rats receiving vehicle treatment, corroborating our microglia morphology data where there were age-associated increases in hippocampal microglia density. Since there were no differences in density following *M. vaccae* immunization, the lowered expression of IBA1 and TMEM119 in the aged hippocampus of rats receiving *M. vaccae* suggests a decrease in microglial activation. Furthermore, cross-sectional human studies using magnetic resonance imaging indicate that there is accelerated and pronounced shrinkage of the hippocampus and cerebellum due to aging compared to regions like the entorhinal and visual cortices^43^.

*M. vaccae* appears to exert brain region-dependent anti-inflammatory properties on microglia. Microglia morphology in the BLA and CA3 hippocampal subfield was most consistently regulated by *M. vaccae* immunization with features indicative of a quiescent phenotype (i.e., decreased density, smaller somas, and increased branching). Our prior work suggests *M. vaccae* immunization diminished age-associated hippocampal neuroinflammation and protected against surgery-induced cognitive decline. Indeed, aged rats that were immunized with *M. vaccae* over three weeks were protected from impairments in memory on a hippocampal-dependent fear conditioning test following surgery. Aged rats that received *M. vaccae* froze ≈ 30% more when presented with the conditioned context relative to their aged counterparts receiving vehicle, suggesting improvements in associative learning and memory following immunization^18^. The CA3 subfield is a major component of the trisynaptic circuit that is responsible for memory consolidation and cognition^44^, and limiting microglia activation in the hippocampus leads to improvements in spatial memory and neurogenesis^45^. Thus, microbial-based therapies using *M. vaccae* may be a promising treatment option to prevent hippocampal activation of microglia and subsequent declines in cognition.

Our microglia morphological results indicate that the amygdala may be another critical region affected by *M. vaccae* immunization. The amygdala is highly responsive to stressful stimuli and can, in turn, modulate the emotional response to stress by promoting fear- and anxiety-related behaviors^46^. Indeed, adult rats immunized with *M. vaccae* had enhanced fear extinction in a fear-potentiated startle paradigm that models post-traumatic stress disorder. This was accompanied by decreases in the expression of corticotropin-releasing hormone, a neuropeptide associated with stress, from the central amygdala^47^. Moreover, *M. vaccae* immunization in mice promotes proactive stress coping behaviors following chronic subordinate colony housing exposure, suggestive of heightened stress resilience. These mice also spent more time investigating the open arms of an elevated plus maze, indicating that *M. vaccae* may have anxiolytic effects^20^.

*M. vaccae* immunization also prevented age-associated increases in microglia density in the BLA but did not alter microglia density in any of the other regions investigated (i.e., CA1, DG, PrL). This is in line with Reber et al. (2016), in which *M. vaccae* immunization did not alter microglia density in eight out of nine cortical, hippocampal, and hypothalamic regions evaluated in adult mice^20^. Our results build off these findings to reveal that even with age-associated increases in microglia density, *M. vaccae* had limited effects on microglia density.

Changes in microglia morphology may be indicative of an imbalance in CNS homeostasis since they are constantly surveilling the brain microenvironment^21^. Indeed, there is a relationship between microglia morphology and their function; for example, upon activation (e.g., injury, infection), there is upregulated expression of pro-inflammatory markers that is concomitant with microglial soma hypertrophy and retraction of processes^48^. However, microglial reactivity is a dynamic and complex process, and differences in microglia morphology do not accurately reflect changes in their release of neuroinflammatory markers. Although this study investigated whether *M. vaccae* immunization protected against age-induced microglia morphological changes, prior work from our laboratory and others supplements our findings and allude to the neuroprotective effects of *M. vaccae*. Aged rats immunized with *M. vaccae* have upregulation of anti-inflammatory *Il4* and *arginase 1* accompanied by downregulated pro-inflammatory *nuclear factor κ B inhibitor alpha*^18^. Moreover, peripheral *M. vaccae* immunization may promote an anti-inflammatory milieu in the CNS by stimulating influx of T cells, suggested by increased hippocampal expression of T cell antigen *Cd3e*^19^.

Overall, it appears that *M. vaccae* affects microglia morphology independent of age. This is not unexpected, as prior work demonstrates *M. vaccae* buffers against stress-induced neuroinflammatory changes in adult rats and mice^19, 20^. *M. vaccae* is thought to signal through modulating T cell populations—depletion of regulatory T cells in mice abolishes the stress-protective effects of *M. vaccae*^20^. Given that adult rats have intact T cell populations^49^, it is possible that *M. vaccae* immunization is more effective at protecting against neuroinflammatory changes in adult rats. T cells numbers decline with age due to thymic involution^49^; however, despite this decrease we detect protective effects of *M. vaccae* in aged rats. For example, there was increased ramification in aged rats microglia following *M. vaccae* administration. This likely represents a beneficial change since there is reversal of the deramification associated with primed microglia in aged rats. In our study, the more limited effects in adult rats on microglia morphology may have occurred due to the lack of an immune challenge. Adult rats were not exposed to a signal that would have increased microglia activation, thus there was immune activation/morphological alterations for *M. vaccae* to “rescue”. Future studies are needed to investigate the effectiveness of *M. vaccae* in adult and aged rats in response to an immune stimulus (e.g., lipopolysaccharide, stress).

Altogether, our study demonstrated that aging and *M. vaccae* immunization had the greatest effect on inducing microglia morphological alterations in the amygdala and hippocampus of F344 × BN rats. Aging caused microglia soma hypertrophy in the CA1 and CA3 subfields that was ameliorated by *M. vaccae* only in the CA3. *M. vaccae* immunization also limited age-related deramification of microglial processes in the BLA and CA3 hippocampal subfield. Collectively, these results indicate that microbial-based approaches may be a promising treatment option for mitigating age-related neuroinflammation and the consequent increased vulnerability to neurological diseases. Future studies should elucidate how peripheral delivery of *M. vaccae* and other anti-inflammatory therapies regulate microglia morphology and phenotype in aged rats.

## Methods

### Animals

Adult (3 months) and aged (24 months) male Fisher 344 × Brown Norway (F344 × BN) F1 rats were used in these experiments. Rats of this age and strain were selected to study healthy, non-neurodegenerative aging since rats of this strain live for more than 30 months and have fewer age-associated pathologies compared to other rodent strains^50, 51^. Rats were received from a National Institute on Aging colony housed at Charles River facility. Colony conditions were maintained at 22 ± 1 °C with a 12:12 light:dark cycle with lights on at 0700 h. Animals were group housed in standard polycarbonate cages on hardwood shavings with access to food (NIH-31) and ultraviolet sterilized, filtered water ad libitum. Upon arrival, rats were pair-housed (52 cm L × 30 cm W × 21 cm H, corn cob bedding, solid bottom) with an age-matched conspecific. Food (Purina rodent chow [5LL2] Prolab RMH 1800) and reverse osmosis filtered water were available ad libitum, and rats were maintained at an ambient temperature of 22 ± 2 °C on a 12:12 light cycle with lights on at 0700 h. Rats were acclimated for at least seven days prior to any experimental manipulation. All experimental protocols were approved by The University of Texas at Austin Institutional Animal Care and Use Committee, following the guidelines of the National Institutes of Health. All experimental procedures were also conducted in accordance with ARRIVE guidelines.

### Experimental design

Adult and aged rats received three subcutaneous injections of whole heat-killed *M. vaccae* *(*NCTC 11,659; 10 mg/mL stock material diluted to 1 mg/mL with sterile borate-buffered saline and 100 μL administered [100 μg M*. vaccae*]) or vehicle spaced one week apart (i.e., one injection per week for three weeks)^18^. Three days following the final injection, rats to be used for microglia morphological analyses were euthanized with a lethal injection of a sodium pentobarbital-containing solution. Once these rats were unresponsive, they were perfused transcardially with saline followed by 4% paraformaldehyde. Their brains were removed and fixed overnight with 4% paraformaldehyde, cryoprotected in 30% sucrose, and frozen at − 55 °C with isopentane on dry ice. Brains were subsequently sectioned onto Fisher Superfrost Plus slides at 18 μm using a cryostat at − 23 °C and stored in a − 20 °C freezer. The subset of rats that were used for gene expression analyses were euthanized with a lethal dose of isoflurane followed by decapitation. Their brains were removed, and the hippocampus, hypothalamus, and prefrontal cortex were dissected. These tissues were then flash frozen in dry ice and stored in a -80 ºC freezer.

### Immunohistochemistry

Prior to immunohistochemistry, slides were blocked with 0.2% Triton X-100 in phosphate-buffered saline containing 10% normal goat serum for one hour at room temperature. Slides were incubated overnight with rabbit anti-Iba1 antibody (1:1,000; Wako; RRID: AB_839504) in blocking buffer at 4 °C, after which they were incubated in biotinylated secondary antibody (1:1,000; Vector Laboratories; Catalog Number: BA-1000-1.5) for one hour and avidin–biotin horseradish peroxidase complex (Vector Laboratories; Catalog Number: PK-6100) for another hour. The sections were then incubated in diaminobenzidine (DAB; Vector Laboratories; Catalog Number: SK-4100) to visualize the activity of the horseradish peroxidase. This reaction was terminated after three minutes when the contrast between the microglia and nonspecific background labeling was optimal. The sections were then dehydrated in a series of alcohols, cleared in xylene, and coverslipped with Permount. Images were captured using an Olympus BX61 bright-field microscope.

### Microglia morphological analyses

Morphological analyses of microglia were performed by a condition-blind observer using FIJI. The various brain regions analyzed were the dorsal hippocampal cornu ammonis 1 (CA1), CA3, and dentate gyrus (DG) subfields (Bregma –3.5 to –4.5 mm), basolateral amygdala (BLA; Bregma –2.0 to –3.0 mm), paraventricular nucleus of the hypothalamus (PVN; Bregma –1.7 to –2.2 mm), and prelimbic cortex (PrL; Bregma + 3.4 to + 4.0 mm).

#### Area fraction analysis

40 × DAB-stained images were batch processed with a macro on FIJI^52^. First, an “FFT Bandpass Filter” was applied to remove background noise smaller than three pixels while maintaining the larger qualities of the image. The image was then converted into an 8-bit grayscale image prior to performing an “Unsharp Mask” filter that sharpens the image's features. A “Despeckle” step was then done to eliminate salt-and-pepper noise. The image was then auto-thresholded, which converts it to a black and white image. A binary “Close” function was performed that connects dark pixels if they are separated by up to two pixels. Lastly, the “Remove Outliers” plug-in was used to further remove background noise up to two pixels in size. The area fraction of the resulting image was then calculated. Each thresholded image was visually inspected to ensure that an accurate area fraction was obtained. If the automatic thresholding failed to remove the background noise, then the thresholding step was manually performed. This analysis had five to six rats per experimental group.

#### Microglia density

Simple morphometric cell counting was performed using the “Cell Counter” plug-in on FIJI. Microglia counts and density were determined by assessing the number of cells and area of staining from 10 × DAB-stained images. Absolute cell count was divided by area analyzed (375,000 pixels^2^ for the BLA; 475,000 pixels^2^ for the CA1, CA3, and PVN; 400,000 pixels^2^ for the DG; and 500,000 pixels^2^ for the PrL) to obtain microglial density. All morphometric analyses were conducted by one trained experimenter, and there were four to six rats per experimental group.

#### Microglial soma characteristics

Microglial somas were traced on FIJI using the “Freehand Selections” tool. A Huion Inspiroy H950P Graphics Drawing Tablet was used for these tracings. 40 × DAB-stained images were utilized, and 12 microglia were analyzed for the BLA, CA1, CA3, DG, PVN, and PrL. Soma area, perimeter, and circularity were then calculated using FIJI. This morphological test had five to six rats per experimental group.

#### Sholl and skeletonization analyses

40 × DAB-stained images from the BLA, CA3, PVN, and PrL were processed by the same macro used for the area fraction analysis. Microglia were isolated, and background noise was removed. The “Paintbrush” tool was then used to fill in gaps between microglial processes. The Sholl analysis was performed using the “Sholl Analysis” plug-in on FIJI. The start radius was defined at five pixels from the center of the microglia soma, and the step size was set to seven pixels. Characteristics of traced microglia were then measured using the “Analyze Skeleton” plug-in function in FIJI. A cutoff value was set at five pixels such that branches shorter than this were excluded from the analysis to avoid capturing small specks of noise that would artificially inflate branching. This plug-in generated values reflecting the total length and branching of microglia processes. Microglia characteristics were evaluated in three to five cells per animal across two to three sections and averaged to generate a single value per animal for statistical comparisons. Images would be captured on sections from either opposing hemispheres or from adjacent brain slices. Only representative microglia that had clear somas and processes were selected and analyzed. Four to six rats per experimental group were utilized for these analyses.

### Gene expression

RNA was extracted from the hippocampus, hypothalamus, and prefrontal cortex by performing a TRIzol-chloroform (Fisher) extraction (as described in Fonken et al., 2016). RNA was then reverse transcribed to cDNA using Superscript IV (Life Technologies) according to the manufacturer’s instructions. PCR amplification of cDNA was performed using TaqMan reagents with a QuantStudio 3 detection system. Gene expression was determined in duplicate and expressed relative to *GAPDH*. There were no group differences in the housekeeping gene. All qRT-PCR results were analyzed using the 2^−ΔΔCt^ method and were normalized such that the control group was set to a value of 1.

### Statistical analyses

Data presented in the figures are expressed as mean ± standard error of the mean (SEM). The α level for all tests was set at 0.05. Statistical analyses consisted of two-tailed two-way analysis of variance (ANOVA; aging × *M. vaccae*) between experimental groups. If there was an interaction effect between aging and *M. vaccae*, Tukey’s post hoc test was performed to correct for multiple comparisons. Normality of residuals was assessed using the D’Agostino-Pearson omnibus K2 normality test. In plotting the relationship among multiple variables (e.g., total branch length vs. soma perimeter; Fig. 5d), the Mahalanobis distance between experimental groups was calculated. This was followed by a χ^2^ test to determine if these groups clustered differently on the XY plane. The analyses and generation of figures were performed using GraphPad Prism 9 and R.

## Supplementary Information

Supplementary Information.

## Data Availability

The datasets generated during and/or analyzed during the current study are available from the corresponding author on request.
