# Supplementary material for: Mycobacterium vaccae immunization in rats ameliorates features of age-associated microglia activation in the amygdala and hippocampus
Source: Sci Rep. 2022 Feb 9;12:2165. doi: 10.1038/s41598-022-05275-y (PMC8828872; doi:10.1038/s41598-022-05275-y)
Supplement: Supplementary file 1 — Supplementary Information. [file 41598_2022_5275_MOESM1_ESM.pdf]

Mycobacterium vaccae immunization in rats ameliorates age-associated microglia activation in the amygdala and hippocampus

Kevin Sanchez[1], Jeffrey S. Darling[1], Reha Kakkar[1], Sienna L. Wu[1], Andrew Zentay[1], Christopher A. Lowry[2,3], and Laura K. Fonken[1*]

[1] Division of Pharmacology & Toxicology, College of Pharmacy, The University of Texas at Austin, Austin, TX 78712, USA

[2] Department of Integrative Physiology, University of Colorado Boulder, Boulder, CO 80309, USA

[3] Center for Neuroscience, University of Colorado Boulder, Boulder, CO 80309, USA

[*] Corresponding author

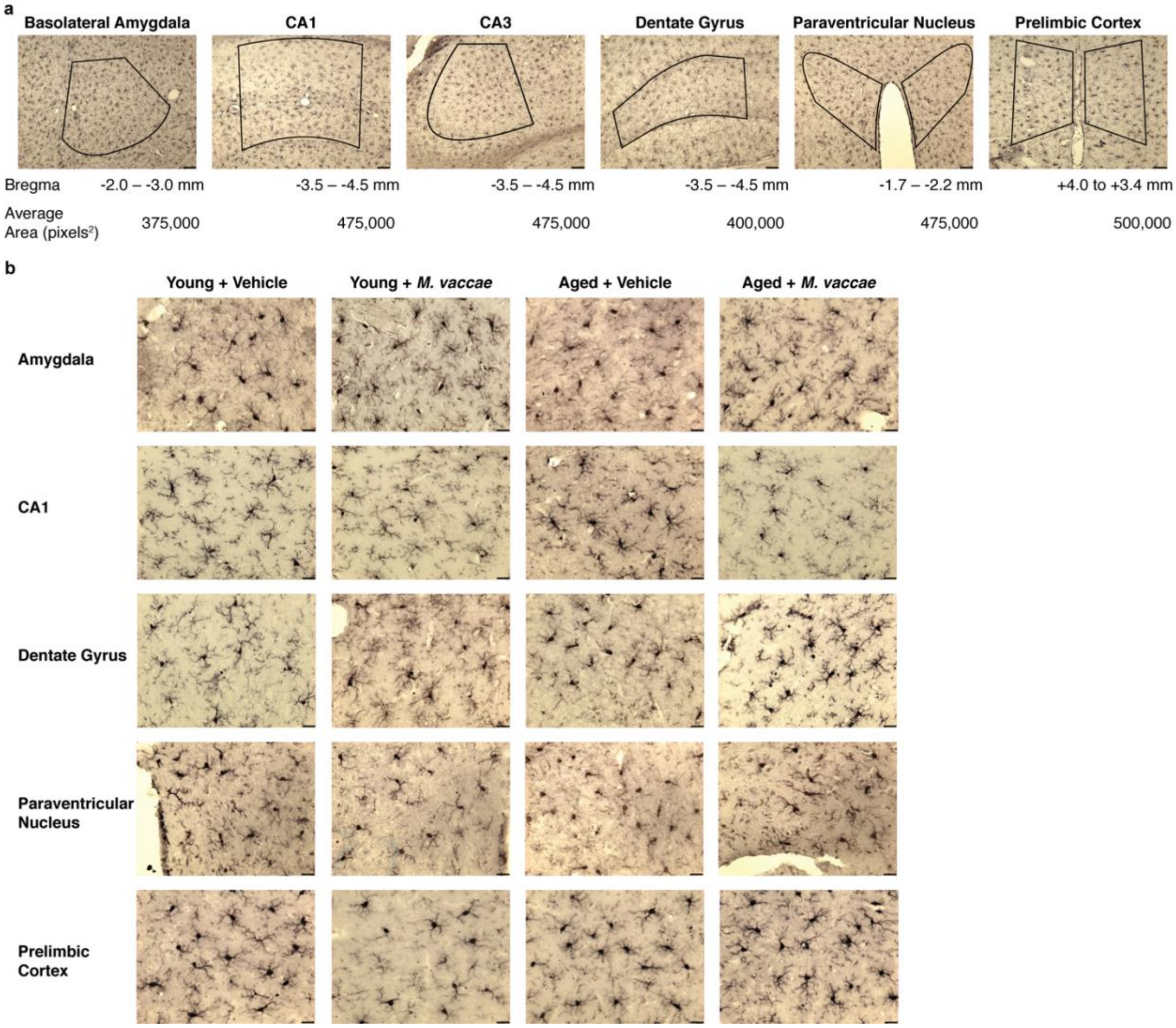

Supplementary Figure S1. Selection of brain regions and representative images. a, Bregma coordinates and average area used in microglia density analysis for the basolateral amygdala, CA1, CA3, dentate gyrus, paraventricular nucleus, and prelimbic cortex. **b,** Representative images of each experimental group for the basolateral amygdala, CA1, dentate gyrus, paraventricular nucleus, and prelimbic cortex. Scale bar = 80 μm (**a**), 20 μm (**b**).
